# Prediction of Range of Motion in Patients After Total Knee Arthroplasty by Shear Wave Elastography

**DOI:** 10.3390/bioengineering12101009

**Published:** 2025-09-23

**Authors:** Min-Woo Kim, Dong-Ha Lee

**Affiliations:** 1 Department of Orthopedic Surgery, Busan Medical Center, Busan 47527, Republic of Korea; drkimminwoo@naver.com; 2 Department of Orthopedic Surgery, Seoul National University College of Medicine, 101 Daehak-ro, Jongno-gu, Seoul 03080, Republic of Korea

**Keywords:** total knee arthroplasty, logistic regression, shear wave elastography

## Abstract

**Introduction**. We hypothesized changes in the elasticity in quadriceps and patella tendon before and after total knee arthroplasty would be correlated with a post-operative range of motion after total knee arthroplasty. To prospectively assess the post-operative range of motion after total knee arthroplasty, logistic regression was adopted with elasticity in the quadriceps and patella tendons were measured using shear wave elastography (SWE). **Materials and Methods**. From March 2021 to June 2021, SWE was performed on 95 patients (86 women; aged 57–85, mean 70.62 ± 5.49 years) preoperatively and 2 days after total knee arthroplasty. Elasticity at quadriceps and patellar tendons were measured with full flexion and extension using SWE. Based on the range of motion after surgery at 56 days, we divided the patients into two groups (Group A > 120 degrees; group B < 120 degrees). Using a logistic regression algorithm, classification between groups was performed. For the input of algorithm, patient information, the elasticity of quadriceps and patella tendons preoperatively and two days after total knee arthroplasty were used. **Results**. The accuracy of predicting group using only patient information was 62%, whereas using only elasticity was 68%. Furthermore, combining information and elasticity before and after surgery at 2 days, accuracy, sensitivity, specificity was 79%, 92%, 56%. **Conclusions**. Combined with patient information, elasticity measured by SWE at pre-op and early post-op periods could be effective to predict the performance of postoperative ROM. This algorithm could provide direction for rehabilitation.

## 1. Introduction

Total knee arthroplasty (TKA) is a widely performed and effective intervention for patients with end-stage knee osteoarthritis. Despite advances in surgical technique and implant design, approximately 11–20% of patients report dissatisfaction after surgery, most commonly due to limitations in postoperative range of motion (ROM) [1,2]. Early prediction of ROM recovery is critical for tailoring rehabilitation protocols and identifying patients at risk of joint stiffness. However, reliable predictors of postoperative ROM in the early phase remain scarce and clinically underutilized.

Shear wave elastography (SWE) is an emerging ultrasound imaging modality that quantifies tissue elasticity in a non-invasive and reproducible manner. SWE has been validated in several clinical contexts, including hepatic fibrosis, breast lesions, and thyroid nodules [3,4]. More recently, its application has expanded into musculoskeletal medicine, including the evaluation of tendon stiffness and healing [5,6]. Preliminary studies suggest that SWE may capture biomechanical changes in soft tissues after orthopedic interventions [7,8]. However, its utility in predicting functional outcomes such as ROM after TKA remains poorly explored and not yet clinically established.

Several studies have investigated anatomical and biomechanical predictors of postoperative ROM. Parameters such as patellar height, joint line position, quadriceps tendon integrity, and preoperative ROM have been associated with outcomes after TKA [9,10,11,12]. Alrefaee et al. demonstrated that radiographic landmarks such as the distance from the fibular head to the joint line correlated with ROM, while other research has highlighted the influence of tendon stiffness and muscle function [10]. Nevertheless, these approaches often rely on postoperative assessments or lack dynamic, real-time evaluation of soft tissue recovery. Thus, the clinical need for an early, objective, and scalable method to predict ROM recovery after TKA remains unmet.

The objective of this study was to evaluate whether SWE-derived measures of quadriceps and patellar tendon elasticity, in combination with clinical variables, can effectively predict postoperative ROM recovery using logistic regression modeling. Establishing such an approach may allow clinicians to identify patients at risk of poor outcomes and implement timely interventions to improve functional recovery after TKA.

## 2. Materials and Methods

### 2.1. Patients

This study was conducted following approval from the Institutional Review Board (Approval No. P01-202104-11-002) of the Public Institutional Bioethics Committee designated by the Ministry of Health and Wellness. Written informed consent was obtained from all participants, including consent for publication of case-related data. All procedures were carried out by a single experienced orthopedic surgeon utilizing either the Persona system (Zimmer Biomet, Warsaw, IN, USA) or the Lospa system (Corentec Inc., Seoul, Republic of Korea). Postoperatively, patients were maintained on absolute bed rest with quadriceps strengthening exercises for the first two days. From postoperative day two, full weight-bearing was permitted, along with continued quadriceps training, range of motion (ROM) exercises, and progressive gait training.

A total of 95 individuals (9 males [9.5%] and 86 females [90.5%]) who underwent 95 total knee arthroplasties (TKA) were enrolled. This provided 475 postoperative tendon measurements (95 patellar tendons and 76 quadriceps tendons) and 475 preoperative tendon measurements (50 patellar tendons and 50 quadriceps tendons) for analysis (Table 1). Patients were excluded if they presented with open wounds, acute infections, periarticular fractures, or comorbid conditions that prevented adequate clinical or ultrasonographic knee evaluation.

### 2.2. Clinical Assessment

All subjects underwent a standardized physical examination performed by an orthopedic specialist. During the clinical visit, patients completed a uniform questionnaire covering their general health history, current analgesic use, previous knee operations, and any prior joint infections. ROM measurements were recorded preoperatively, and again at 2 days, 4 weeks, and 8 weeks following TKA.

### 2.3. Elasticity Measurement by SWE

Ultrasound of knee was performed on patients who underwent TKA in our hospital, and the tissue elasticity was measured using SWE for preoperative and postoperative periods (2 days after TKA). Elasticity was measured in rectus femoris, vastus medialis, vastus lateralis, patella tendon, and lateral biceps tendon, and the elasticity of these tissues were measured for maximum flexion and maximum extension, respectively. Elasticity among tissues shown on ultrasound was measured where the RMI was above 0.7 and the measured values were shown even. According to manufacturer’s instruction and literature [13,14]. SWE elasticity values were recorded only when the displayed values were stable and met the RMI threshold [15].

### 2.4. Prediction of Postoperative ROM

This study was designed as an exploratory, prospective pilot analysis; thus, a formal power calculation was not performed a priori. However, based on post hoc estimation using a two-group logistic regression model with α = 0.05, our sample size (*n* = 95) provides approximately 78% power to detect a moderate effect size (Cohen’s d ≈ 0.5). Future studies with larger cohorts will be needed for confirmatory validation.

From the medical records, patient-specific parameters including hypertension (HTN), diabetes mellitus (DM), preoperative ROM, age, and sex were extracted. Although elasticity was measured in rectus femoris, vastus medialis, vastus lateralis, patella tendon, and lateral biceps tendon, only quadriceps and patella tendon values were used for analysis.

Elasticity measurements were obtained for 5 different tissues (rectus femoris, vastus medialis, vastus lateralis, patellar tendon, and lateral biceps tendon) in 2 knee positions (flexion and extension) at 2 time points (preoperative and postoperative day 2), resulting in a total of 20 measurements per patient.

Patients were categorized based on ROM at 8 weeks (POD56), but prediction performance was evaluated at both 4 and 8 weeks postoperatively to assess short-term and mid-term predictability. Logistic regression analysis was conducted using a total of 25 parameters to predict postoperative ROM. Patients were categorized into two groups: Group A (ROM > 120°) and Group B (ROM < 120°). The predictive performance of the model was assessed using accuracy, sensitivity, and specificity metrics. A 120° threshold was chosen to define postoperative ROM groups, consistent with previous studies that used 110–125° as clinical indicators of functional knee flexion after TKA. Flexion >120° is generally associated with the ability to perform common activities such as squatting and stair climbing [16,17].

To evaluate the independent contribution of SWE measurements, an additional logistic regression model was developed using only patient demographic and clinical information. The same evaluation metrics were calculated to determine the incremental benefit of SWE integration.

In logistic regression (LR), the probability of a patient belonging to Group A or Group B is estimated using a log-odds formulation:

$$z_{j}=\;w_{0}+\;\sum_{i=1}^{25}w_{i}x_{ij}$$

where $z_{j}$ is the log-odds for the $j^{th}$ patient, $w_{0}$ is the intercept, $w_{i}$ represents the coefficient for the $i^{th}$ parameter, and $x_{ij}$ denotes the $i^{th}$ feature value for the $j^{th}$ patient. The log-odds are transformed into probabilities between 0 and 1 via the sigmoid function $f_{\mathrm{sig}}\left(\cdot \right)\;$. Coefficients are optimized using the cross-entropy loss function:

$$\left({\hat{w}}_{0},\;{\hat{w}}_{1},...,{\hat{\omega }}_{25}\;\right)=\;arg\underset{w_{0},\ldots ,w_{25}}{min}\left\{-\frac{1}{N}\sum_{j=1}^{N}\left[y_{j}\log_{\sigma }(z_{j})+(1-y_{j})\log(1-\sigma (z_{j}))\right]\right\}$$

where $y_{j}$ is the binary class label (Group A = 1, Group B = 0) and NNN is the number of patients. Once optimized, the probability for each patient is computed as:

$$p_{j}=f_{sig}\;\left({\hat{w}}_{0}+\;\sum_{i=1}^{25}w_{i}x_{ij}\right)$$

Patients were classified using a default threshold θ= 0.5 (Group A if $p_{j}>\theta$; Group B otherwise). Threshold variation was applied to generate the receiver operating characteristic (ROC) curve and determine the optimal cut-off. All LR procedures were implemented using the ‘mnrit’ and ‘mnrval’ functions in MATLAB 25.1 [18].

## 3. Results

### Patient Characteristics

The overall demographics are shown in Table 1. A total of 95 patients (9 males and 86 females) were included in the final analysis. Their mean age was 70.62 ± 5.49 years. Overall, there was no statistically significant difference between the two groups in terms of age, sex, or hypertension. However, the prevalence of diabetes mellitus was significantly higher in Group B (*p* = 0.041).

In predicting ROM in the 4 weeks and 8 weeks after surgery, (1) a model using only the patient’s basic information and preoperative ROM, (2) a model using only the SWE values of the preoperative and second days after surgery (3) All values of (1) and (2) were made. The accuracy, sensitivity, and specificity of the predicted model are shown in Table 2.

The model using only SWE performed better accuracy, sensitivity, and specificity than when only the patient’s basic information and preoperative ROM were used for.

To measure the performance of the model objectively, receiver operating characteristic (ROC) curves were calculated. (Table 3) ROC Curve was created by calculating sensitivity and specificity while gradually changing the threshold for each model. Based on ROC curve, AUC (Area Under ROC Curve) was calculated. Like the previous results, the AUC value was higher in the model calculated using only SWE than the model calculated using only patient information on both 28 and 56 days after surgery. Furthermore, the combined model showed the highest AUC value, indicating superior predictive performance [19].

## 4. Discussion

In this shear wave elastography study on quadriceps and patellar tendons following TKA, we were able to demonstrate importance of shear wave elastography on predicting range of motion after TKA [20,21]. In recent research, SWE reflects changes the course of tendon healing and is reliably able to measure and display these tendon changes, which, in line with previous studies [22,23].

In recent years, SWE has been increasingly used to better understand the mechanical properties of tendons as well as to monitor the healing process in tendinopathies. An excellent interobserver, intraobserver, and retest reliability for the quadriceps Dirrichset Et al. recently confirmed this in a double-blinded and the patellar tendon could be verified. Study for tendinopathies and were able to show that this method is superior to B-US and PD-US [24].

In this study, the elasticity of the quadriceps/patella tendon were measured before and 2 days after surgery using the characteristics of SWE [25]. After that, combining with the patient’s medical history and preoperative range of motion, ROM at 4 weeks and 8 weeks postoperatively were predicted using logistic regression. It has been shown that SWE is useful in predicting postoperative ROM in patients.

While typical studies so far have analyzed how each factor affects ROM individually, this study analyzes whether each factor has a complex effect [26,27,28]. It was found that the overall quadriceps tendon values, where no single variable was important, influenced the ROM [29,30].

In addition, sensitivity, specificity, accuracy results of predicting postoperative ROM (1) only using the factors that have been commonly known so far, (2) quadriceps elasticity measured using SWE, it was found that (2) had better results than (1). Based on this, we could confirm that the measured value of SWE can help predict ROM after TKA. Moreover, more accurate predictions can be made if the models presented in this study are mixed with the existing factors.

Although early predictions of ROM can guide rehabilitation strategies, we recognize that ROM improvement continues beyond the 8-week period assessed in this study. Future studies with 3- to 6-month or longer-term follow-up are necessary to evaluate the predictive validity of SWE over a more extended rehabilitation timeline. Our study cohort consisted predominantly of female patients (90.5%), reflecting the higher prevalence of advanced knee osteoarthritis among elderly women. Nevertheless, this imbalance may limit the generalizability of our findings to male populations and warrants caution in interpretation.

Also, a key limitation of our model is the absence of a separate validation cohort. While the current analysis provides preliminary insight, future work should employ k-fold cross-validation and external validation with larger multicenter datasets to enhance generalizability. To reduce inter-operator variability, all SWE measurements were conducted by a single experienced orthopedic surgeon. While this increases measurement consistency, future studies should assess inter-rater and intra-rater reliability for broader applicability. Passive range of motion (ROM) was measured using a standard goniometer by a single orthopedic surgeon with over 5 years of clinical experience. All measurements were performed under standardized conditions with patients in the supine position, ensuring consistency throughout the study.

Therefore, it is thought that the results in this paper can help predict ROM on 2 months after TKA early. Based on this, it is thought that it will be possible to give options to consider active intervention in post operative care and rehabilitation plan establishment.

Although the combined model demonstrated improved overall accuracy (79%) and high sensitivity (92%), the relatively low specificity (56%) suggests a risk of overestimating functional recovery in some patients. In clinical terms, this means certain patients at risk of limited ROM may be misclassified as low-risk and not receive early rehabilitation interventions. Future work should focus on refining the model to optimize both sensitivity and specificity, possibly by incorporating additional predictive variables or machine learning techniques.

Combined with patient information, elasticity measured by shear wave elastography at pre-operative and early post-operative period could be effective to predict performance of postoperative ROM. This algorithm could provide direction for rehabilitation.

## 5. Conclusions

This study demonstrated that SWE measurements of quadriceps and patellar tendon elasticity, when combined with basic patient characteristics, can effectively predict postoperative ROM after TKA. Although limitations such as short follow-up and moderate specificity exist, these findings highlight the clinical value of SWE as a supportive tool in early rehabilitation planning.

## Figures and Tables

**Table 1:** Demographic data of patients.

	Total	Group A (ROM > 120)	Group B (ROM < 120)
Case (number)	95	61	34
Mean age (years)	70 ± 5	70 ± 5	70 ± 5
Sex (Male/female)	86/9	56/5	30/4
Average ROM	122 ± 12	123 ± 12	121 ± 13

**Table 2:** Performance for prediction ROM at 4 weeks and 8 weeks after TKA using model with patient basic information, SWE value and both information, respectively.

	Patient Basic Info POD28	SWE Only POD28	All POD 28	Patient Basic Info POD56	SWE Only POD56	All POD 56
Accuracy	56/96 0.58	63/96 0.65625	73/96 0.76	59/95 0.62	65/95 0.68	75/95 0.79
Sensitivity	18/43 0.42	24/43 0.56	31/43 0.72	50/61 0.82	51/61 0.84	56/61 0.92
Specificity	38/53 0.72	39/53 0.74	42/53 0.79	9/34 0.26	14/34 0.41	19/34 0.56

**Table 3:** AUC value after logistic regression. For each model, AUC was calculated by changing threshold.

	Patient Basic Info POD28	SWE Only POD28	All POD 28	Patient Basic Info POD56	SWE Only POD56	All POD 56
AUC	0.66	0.70	0.80	0.68	0.74	0.78

## Data Availability

The original contributions presented in this study are included in the article. Further inquiries can be directed to the corresponding author.

## Abbreviations

TKA: Total Knee Arthroplasty; SWE: Shear Wave Elastography; ROM: Range of motion; RMI: Reliability of the measurement; LR: Logistic regression; ROC: Receiver operating characteristic; AUC: Area Under ROC Curve.
